# Multi-Step Structure Image Inpainting Model with Attention Mechanism

**DOI:** 10.3390/s23042316

**Published:** 2023-02-19

**Authors:** Cai Ran, Xinfu Li, Fang Yang

**Affiliations:** 1 School of Cyber Security and Computer, Hebei University, Baoding 071002, China; 2 Machine Vision Engineering Research Center, Hebei University, Baoding 071002, China

**Keywords:** image inpainting, image reconstruction, generative adversarial networks, deep learning

## Abstract

The proliferation of deep learning has propelled image inpainting to an important research field. Although the current image inpainting model has made remarkable achievements, the two-stage image inpainting method is easy to produce structural errors in the rough stage because of insufficient treatment of the rough inpainting stage. To address this problem, we propose a multi-step structured image inpainting model combining attention mechanisms. Different from the previous two-stage inpainting model, we divide the damaged area into four sub-areas, calculate the priority of each area according to the priority, specify the inpainting order, and complete the rough inpainting stage several times. The stability of the model is enhanced by the multi-step method. The structural attention mechanism strengthens the expression of structural features and improves the quality of structure and contour reconstruction. Experimental evaluation of benchmark data sets shows that our method effectively reduces structural errors and improves the effect of image inpainting.

## 1. Introduction

Image inpainting is a technology that fills the damaged area of an image into a complete image that conforms to human visual effects and cognition. With the development of deep learning technology, image inpainting has become an important research field. Image inpainting has been widely used in art restoration, special effects production for film and video, image editing, and other fields. Nowadays, image inpainting is mainly divided into two schemes: the traditional mathematical method and the deep learning method based on convolutional neural networks. Traditional image inpainting methods mostly use mathematical calculation, a non-learning method, to extract the complete information of the image and fill in the damaged area of the image. This method cannot extract the deep information of the image, which leads to the lack of semantic information in the synthesized image, resulting in abrupt restoration results which deviate from human visual perception. Especially when inpainting images with large holes, traditional inpainting methods can easily lead to failure. Because the larger the hole in the image, the more complex the internal texture, structure, and semantic information.

More recently, convolutional neural networks (CNN) in deep learning can alleviate the above problems. The deep information of the image can be better extracted by convolution calculation, and the generated image information is more abundant. Ian Goodfellow proposed the generative adversarial networks(GAN) [1] in 2014. Since then, the fields related to image generation [2,3,4] have witnessed significant development. The confrontational training mode of the network can make the network generate more realistic images. GAN is mainly composed of a generator and discriminator, both of which are composed of convolutional neural networks. The generator is responsible for generating the repaired image, then inputting the restored image and the raw image into the discriminator for identification, and feeding the identification result back to the generator. In this confrontation training process, the image generated by the generator is gradually colorful, the texture details are more abundant, and the discriminator is gradually improving its ability to distinguish the synthetic image from the natural image. To sum up, GAN is undoubtedly the most promising method for deep inpainting.

Presently, GAN-based depth image inpainting methods are divided into two directions, one-stage network and two-stage network, and their difference lies mainly in the generator. The single-stage network is primarily an end-to-end model, and the generator directly outputs the restoration image. Recently, the Shift-net proposed by Yan et al. [5] adopts a single-stage network model. By combining the shift connection layer with U-Net, for filling in missing regions of any shape with sharp structures and fine-detailed textures. This approach still fails to address the issue of indistinctness in the single-stage network model, resulting in the loss of texture details in the generated image. In subsequent work, Liu et al. [6] proposed the MED model to balance the texture and structural features with the aim of preserving both in the inpainting results.

Most of these single-stage depth image inpainting methods lack the processing of image details, resulting in overly smooth synthesized images with blurred images and a lack of texture and structural information. Two-stage generator network adopts more methods to enhance image details. This structure is like a painter creating, which first generates the structure information or sketch of the image and generates the refined image in the second stage. Yu et al. [7] proposed a Contextual Attention Model that roughly restores the entire image based on the context in the initial stage and proceeds to generate more refined results in the subsequent stage. Nazeri et al. [8] introduced the EdgeConnect Model that prioritizes restoring the image’s outline in the first stage, and then completes the colorization in the second stage.

Two-stage generators can often express more vivid textures and semantic information. However, the coarse-to-fine network depends on the image restoration result of the first stage. Suppose the first stage produces a coarse image with some deviations. In that case, the second stage has a finely filled effect that deviates from human visual perception, with obvious structural and semantic errors.

To improve the reliability of the coarse-to-fine image inpainting algorithm and to address the lack of image structure processing in the coarse-to-fine network model, we propose a coarse-to-fine deep image inpainting model based on the multi-step structure inpainting model. Our proposed network focuses on the coarse stage of the restoration, as this is the basis for the final image restoration. The coarse-to-fine generator adopts the Unet structure and uses the skip connection operation to counter the gradient disappearance problem and improve the transmission ability of the image information in the deep network. In the first stage, we input the contour information of the image into a one-stage network to reconstruct the structural features of the image. Unlike previous deep fill methods, we do not rebuild the damaged area at once but inpainting it in steps. We divide the occlusion area into four sub-areas and then gradually restore it. At present, the attention mechanism is widely used in various fields [9,10,11,12]. Attention mechanisms can better extract the important content of the image and improve the feature expression ability of the network. Therefore, we introduced a structural attention module to improve the ability to extract structural information. In this attention module, the local binary pattern(LBP) operator and grayscale images enhance structural features, and gated convolution is used to extract features. Finally, the enhanced feature information is transmitted to the decoder. We added local and global discriminators to the coarse-to-fine network to improve image inpainting’s details and overall effect. In particular, in the second stage of the discriminator, we additionally use perception loss and style loss to enhance the authenticity. Experimental results demonstrate that our method effectively enhances the structural reconstruction of the two-stage inpainting model and minimizes the occurrence of incorrect structures.

The main contributions of this paper are as follows:

(1) We propose a coarse-to-fine inpainting network, in which we inpaint the structural information of the image in the first stage and color the reconstructed area in the second stage. At the same time, to solve the problem that the damaged area is challenging to recover, we put forward a multi-step structure inpainting scheme.
(2) We introduce a structural information attention module to improve the ability to reconstruct structural information in the first stage.
(3) Our proposed method outperforms existing methods in the benchmark dataset. It resolves the instability issue of two-stage models, such as the gated convolution model [13] and Edgeconnect model [8], in image structure reconstruction, resulting in improved restoration effects. Our method demonstrates better inpainting results compared to MED [6], a single-stage restoration model.

## 2. Related Work

Advances in image inpainting technology have made it possible to perform these tasks with greater precision and accuracy, resulting in more visually appealing and useful images. This has led to a growing demand for image inpainting solutions in a variety of industries, including photography, printing, and multimedia. In addition, image inpainting is becoming an important branch in the field of privacy protection [14,15,16,17,18]. We briefly reviewed the work related to this paper.

### 2.1. Traditional Mathematical Method

The traditional mathematical method uses non-learning techniques to fill in images before the deep learning technology matures. This kind of method is mainly based on the idea of diffusion filling and block matching.

The diffusion-based method spreads the boundary of the complete area to the damaged area to complete the filling. At first, Bertalmio M et al. [19] proposed a diffusion method based on a partial differential equation by combining the idea of art restoration. Later, methods based on partial differential equation diffusion include total variation model [20], Euler’s elastica model [21], M um ford Shah model [22], etc. The diffusion-based operation has a good effect on restoring small missing areas like scratches, but it can not complete the large damaged images.

The method based on patch matching is to search the patch from the complete area of the image to complete the filling. This method can fill some large damaged areas more naturally than diffusion-based technology. However, the block matching method cannot fill the image that conforms to human vision. The filled image lacks semantic information, so it cannot generate an image that fits human cognition. Drori et al. [23] iterated through the missing area content by using the smooth interpolation method and filled the most similar image blocks into the occlusion area after adding details transformation. Criminisi et al. [24] adopted priority to calculate the restoration order and globally matched similar regions for filling, thus preferentially ensuring the propagation direction of the structural texture. The Criminisi algorithm can restore some images with simple texture structure but still cannot deal with complex content. Wilczkowiak et al. [25] can make guided fixes interactively with the user by searching for similar patch areas. Although these approaches outperform diffusion-based approaches in occlusion inpainting, they still fail to fill in more complex textures and structures, especially lacking semantic information.

### 2.2. Image Inpainting Based on Deep Learning

After Goodfellow et al. [1] put forward the Generative Adversarial Networks (GAN), Pathak et al. [26] introduced GAN into the field of image inpainting for the first time and put forward the Context Encoders model. The generator adopts the structure of an encoder and decoder. Context encoders model uses a convolution neural network, which can inpaint the semantic information of the image and a large occlusion area. However, the fully connected network employed between the encoder and decoder of the context encoder network results in the image being too smooth and fuzzy. After that, to enhance the texture and structural details, Iizuka et al. [27] used the fully convolutional generator and added the local discriminator to form the global and local image inpainting model. However, the double discriminator model still lacks the processing of image details. Yang et al. [28] proposed a joint generation structure to constrain the image content and texture. Yan et al. [5] constructed a single-stage generator network based on Unet network and introduced a shift connection layer to fill in any missing shape area with sharp structure and delicate texture. However, the single-stage Unet network structure still causes too smooth reconstruction results. Yu et al. [7] constructed the coarse-to-fine generator architecture, put forward the context attention mechanism, further ensured the consistency between the inpainting area and the complete area, and adopted the improved Wasserstein gan [29] stability training. Liu et al. [30] proposed partial convolution to improve the inpainting effect of irregular area occlusion. Yu et al. [13] improved the partial convolution, presented a learnable gated convolution, and allowed users to edit images interactively. Inspired by artistic creation, Nazeri et al. [8] adopted the coarse-to-fine network structure to restore the outline information of the image in the first stage and finish the delicate filling of the image in the second stage. However, in the first stage, the image contour is reconstructed at one time, so it is prone to cause the wrong outline, which eventually leads to the failure of the second stage. Liu et al. [31] designed a coherent semantic attention mechanism to strengthen the relationship between missing regional features. Liu et al. [6] think that coarse-to-fine generators often cause serious semantic errors, so they put forward a joint encoder-decoder model and introduced feature equalization operation. However, the joint encoder-decoder model still cannot completely solve the image-blurring problem generated by the single-stage model. More recently, Zhu et al. [32] proposed mask-aware dynamic filtering (MADF) and used a cascade thinning network to fill in any missing image area. Wu et al. [33] used the stack network to repair the occluded image from coarse to fine, improving the reusability of the extracted features.

In the depth inpainting model, the disadvantages of the single-stage model [34,35,36,37,38,39], such as the lack of texture details of the generated image, the fuzzy structural problem still cannot be effectively solved. Most coarse-to-fine models [40,41,42,43,44,45,46] can produce more realistic textures, so many researchers are committed to improving the coarse-to-fine image inpainting model. However, they lack details in the rough repair stage. Moreover, most coarse-to-fine networks use the one-time restoration of all damaged areas, so if the first stage produces an inaccurate image, this error will be amplified in the subsequently refined networks and ultimately creates an unreasonable image. These problems can lead to image inpatient instability, sometimes producing compelling images and sometimes severe semantic errors.

## 3. Approach

### 3.1. Model

To solve these problems in the coarse-to-fine model, we propose a two-stage image inpainting model, which focuses on restoring structure and outline in the first stage because it is the cornerstone of texture and color filling in the fine inpainting stage. We propose a multi-step structure inpainting model to improve the first-stage restoration effect and reduce the difficulty of structural reconstruction. Specifically, in the coarse inpainting stage, we divide the occluded area of the image into four regions, determine the inpainting order by priority calculation, and then input the first-stage network to complete the inpainting gradually. The purpose of this is to restore the most straightforward part in each step, and then when rebuilding the rest, the occluded area can sample more surrounding information to complete the filling. We all know that GAN is difficult to train, prone to model collapse, and challenging to converge. The occlusion area is divided into multiple fills, reducing the difficulty of the inpainting and reducing the pressure of the generator, thus improving the inpainting effect. In addition, we propose a structural attention mechanism to enhance the reliability of structural reconstruction. The attention mechanism uses the gray-scale and LBP images of the damaged image as input, fills the holes with six consecutive layers of gated convolution, and then sends the feature information to the decoder at each scale to complete the feature information enhancement. Then, the first-stage reconstructed contour image and the original image are input into the fine-stage network, and the color filling of the image is completed through Unet to obtain the final restored image.

**Figure 1:**
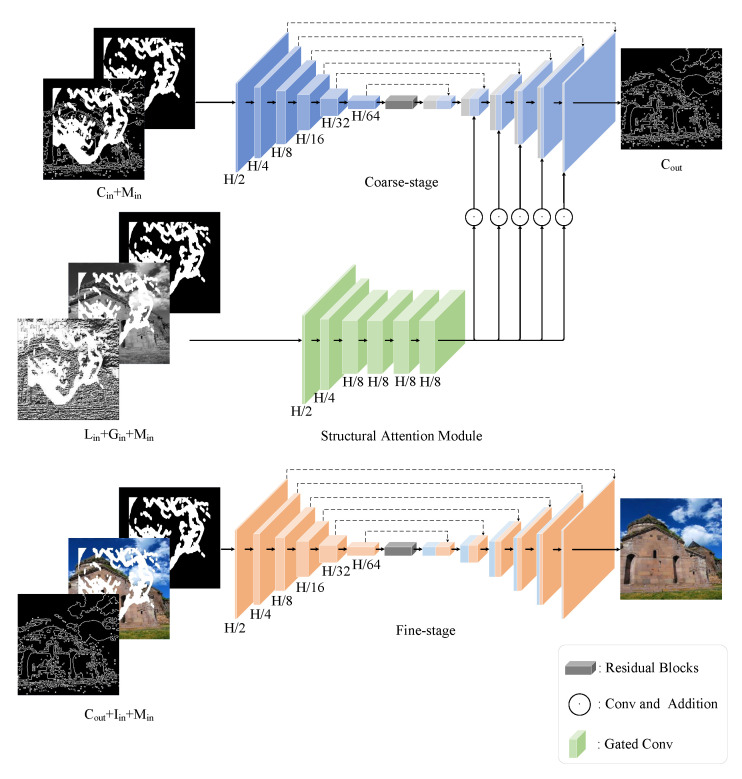
Generator structure diagram. The upper part is the coarse inpainting stage and structural attention module, and the lower part is the fine inpainting stage. It should be noted that the final reconstructed image in the coarse inpainting stage is the result of four rounds reconstruction.

The proposed network structure is shown in Figure 1. For the convenience of explaining the network structure, this figure omits the gradual inpainting stage and only shows the initial input and final inpainting results.

As shown in Figure 1, let $C_{in}$∈R${}^{H}$${}^{\times }$${}^{W}$${}^{\times }$${}^{1}$ be the outline of the damaged image. Let $M_{in}$∈R${}^{H}$${}^{\times }$${}^{W}$${}^{\times }$${}^{1}$ be the original mask. Let $L_{in}$∈R${}^{H}$${}^{\times }$${}^{W}$${}^{\times }$${}^{1}$ be the LBP characteristic map of the damaged image. Let $G_{in}$∈R${}^{H}$${}^{\times }$${}^{W}$${}^{\times }$${}^{1}$ be the gray scale of damaged image. In the backbone network of the rough inpainting stage, we use the contour image $C_{in}$ and mask $M_{in}$ of the damaged image for reconstruction. In the structural attention module, the input is the LBP feature map $L_{in}$ and grayscale map of the damaged image $G_{in}$, and the mask $M_{in}$ to extract the feature information. The backbone network in the rough inpainting stage adopts the Unet structure. The first layer of convolution in the encoder will downsample the image $C_{in}$ to $\frac{H}{2}\times \frac{W}{2}\times 16$ dimension feature map, the second layer will downsample to $\frac{H}{4}\times \frac{H}{4}\times 32$, and the subsequent three-layer convolution network will gradually downsample to $\frac{H}{64}\times \frac{W}{64}\times 512$ dimension. Moreover, our decoder is symmetrical to the encoder, which ensures that the feature information is not lost in the image decoding process. The jump connection is introduced to prevent gradient disappearance caused by deep network structure effectively. At the connection between the encoder and decoder, we do not adopt the traditional full connection operation because that will cause image blur. Specifically, we use a residual block composed of three layers of residual convolutions to connect the encoder and the decoder. Compared with the fully connected layer, the convolutional layer can better express the image features. At the same time, the residual structure can effectively solve the problem of gradient disappearance caused by the deep network.

In the proposed structural attention module, we use the LBP feature map. LBP operator has good feature extraction ability, and undistorted illumination, which solves the problem that features information is difficult to extract due to uneven illumination of images. The equation for the LBP operator is as follows:

(1) $$I_{LBP(x_{c},y_{c})}=LBP\left(x_{c},y_{c}\right)$$

where $\left(x_{c},y_{c}\right)$ represents pixel points, $LBP(x_{c},y_{c})$ denotes the feature extraction of corresponding coordinate points, which is expressed as:

(2) $$LBP\left(x_{c},y_{c}\right)=\sum_{p=0}^{p-1}2^{p}s\left(i_{p}-i_{c}\right)$$

where *p* is the neighborhood pixel, *c* is the center pixel, and *s* is the symbolic function, which is expressed as:

(3) $$s\left(x\right)=\left\{\begin{array}{cc} 1 & x\geq 0\\ 0 & x<0 \end{array}\right.$$

The attention module uses six-layer gated convolution, the gated convolution operation is expressed as:

(4) $$G_{x}=\phi \left(X\cdot W+b\right)⊗\sigma \left(X\cdot V+c\right).$$

We utilize *W* and *V* to denote different convolution filters. ϕ represents the ELU activation function, which is used to process the feature vector. Furthermore, σ represents the sigmoid activation function, which activates the gating operation and maps the gating value between 0 and 1.

The attention module also adopts a six-layer convolutional network, so that the dimension of the feature map becomes $\frac{H}{8}\times \frac{W}{8}\times 64$, which is convenient for upsampling and combining the structural feature information with the decoder.

In the fine-stage, let $I_{in}$∈R${}^{H}$${}^{\times }$${}^{W}$${}^{\times }$${}^{3}$ be the color damaged image. $C_{out}$∈R${}^{H}$${}^{\times }$${}^{W}$${}^{\times }$${}^{1}$ be the outline image of coarse-stage output. Finally, $I_{in}$, $C_{out}$, and $M_{in}$ are input into the fine-stage network to finish the final image coloring. The generator model used in the fine inpainting stage is consistent with the coarse inpainting stage.

### 3.2. Multi-Step Structure Inpainting

**Figure 2:**
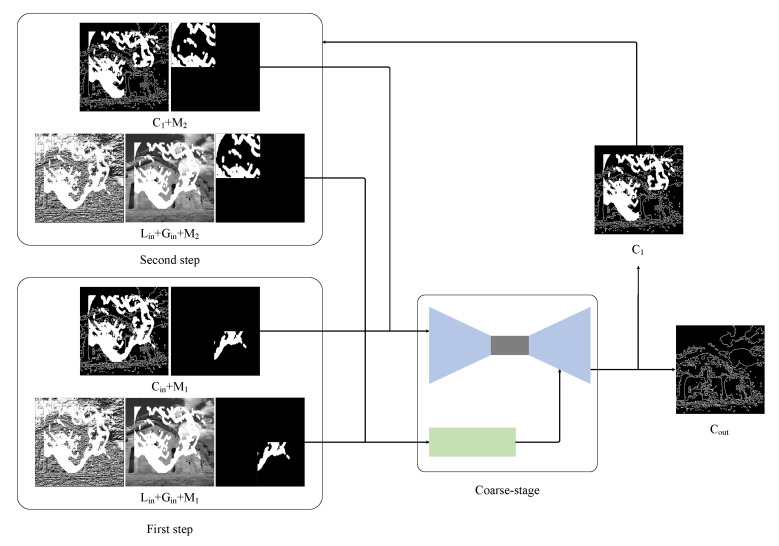
Multi-step structure model flowchart. The rough inpainting stage prioritizes structural reconstruction, and therefore, gray-scale images are used as input. The occlusion area is restored in four steps, with the inpainting order determined by priority calculation. The inpainting process begins with the lower right corner area, which has the highest priority, followed by the upper left corner area with the second highest priority. The final two steps follow a similar pattern. The result in the rough inpainting stage is represented as $C_{out}$.

The specific process of the coarse inpainting stage is shown in Figure 2. We divide the mask into four parts and determine the inpainting order according to the priority policy. The proposed priority calculation method is shown in Equation (1). We determine that priority order according to the size of the complete area contained in each part, let $pri_{i\in 1,2,3,4}$ denote the priority of the *i*-th block mask, $\sum_{j}D_{i,j}$ denote the total number of damaged pixels in the *i*-th mask area, and $\sum_{k}C_{i,k}$ denote the total number of complete pixels in the *i*-th mask area. The higher the pri value, the higher the priority. We prioritize inpainting high-priority occlusion blocks due to their smaller masked areas and greater ease of restoration. By first reconstructing high-priority areas, there will be adding reference information available when restoring low-priority areas, leading to better inpainting results and fewer structural errors.

(5) $$pri_{i\in 1,2,3,4}=\frac{\sum_{j}D_{i,j}}{\sum_{k}C_{i,k}}.$$

We show the first two steps of the multi-step structure inpainting model in Figure 2. In the first step, let $M_{1}\in R^{H\times W\times 1}$ denote the first block of the occlusion area that needs to be restored. Input $C_{in}$, $M_{1}$ into the backbone network of the coarse-stage, and input $L_{in}$, $G_{in}$, $M_{1}$ into the structural attention module. Then the contour image $C_{1}$ reconstructed in the first step is obtained. In the second stage of inpainting, input $C_{in}$, $M_{1}$ into the backbone network of the coarse-stage, and input $L_{in}$, $G_{in}$, $M_{1}$ into the structural attention module. After four restoration times, the final contour restoration image $C_{out}$ is obtained. Finally, the image inpainting is completed through the fine-stage network.

**Figure 3:**
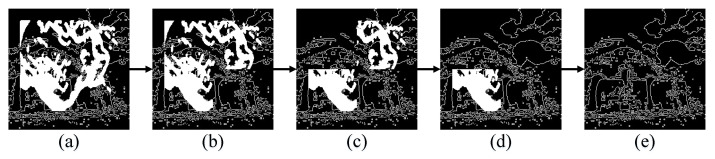
Multi-step structural reconstruction process. (**a**) represents a masked image, (**b**–**d**) depict the inpainting results from the first to third steps, and (**e**) shows the final structural reconstruction result.

The restoration process of an image is shown in Figure 3. Based on the priority calculation, the lower right corner area has the highest priority due to its smallest occluded area, so it is restored first. In the second step, the inpainting result (b) is passed to the coarse inpainting model for reference during the second step of inpainting. While the last area has the lowest priority and the largest occluded area, the use of reference information from the previous three steps reduces inpainting difficulty, improves generator stability, and enhances the structural reconstruction effect.

### 3.3. Loss Function

According to the different functions of the coarse inpainting network and fine inpainting network, we use two joint loss functions to complete the training of the network.

The coarse-stage network does not contain color, style, and texture features, so we only use feature-matching loss, pixel-level reconstruction loss, and adversarial loss to constrain the structural reconstruction of the image. In the fine-stage network, we use pixel-level reconstruction loss, adversarial loss, perceptual loss, and style loss to constrain jointly.

#### 3.3.1. Feature-Matching LOSS

Similar to Edgeconnect [8], we used Feature-matching loss to guarantee the reconstruction effect of the structure image.

(6) $$L_{fm}=E[\sum_{i=1}^{L}\frac{1}{N_{i}}\|D_{1}^{\left(i\right)}\left(C_{gt}\right)-D_{1}^{\left(i\right)}\left(C_{pred}\right){\|}_{1}].$$

where *L* is the final convolution layer of the discriminator, $N_{i}$ is the number of elements in the *i*’th activation layer, and $D_{1}^{\left(i\right)}$ is the activation in the *i*’th layer of the discriminator.

#### 3.3.2. Reconstruction Loss

To improve the refinement at the pixel level of the image, we use L1 distance as the reconstruction loss to measure the error between the predicted image and the actual image.

(7) $$L_{rec}={\left\|I_{out}⊗\left(1-M_{in}\right)-I_{gt}⊗\left(1-M_{in}\right)\right\|}_{1}.$$

$I_{out}$ represents the restored image and $I_{gt}$ is the real image. $M_{in}\in 0,1$ is the input mask, where 0 represents the mask area, 1 represents the complete area, and represents element-by-element multiplication.

#### 3.3.3. Adversarial Loss

According to the characteristics of network training, we utilize relativistic average LS adversarial loss [6] to stabilize the training of GAN.

(8) $$\begin{array}{c} L_{adv}=-E_{I_{gt}}\left[log\left(1-D_{ra}\left(I_{gt},I_{out}\right)\right)\right]\\ \;\;\;-E_{I_{out}}[log\left(D_{ra}\left(I_{out},I_{gt}\right)\right]. \end{array}$$

where $D_{ra}\left(\cdot \right)$ is defined as:

(9) $$D_{ra}\left(I_{gt},I_{out}\right)=sigmoid\left(C\left(I_{gt}\right)-E_{I_{out}}\left[C\left(I_{out}\right)\right]\right).$$

where C(·) indicates the discriminator without the last sigmoid function.

#### 3.3.4. Perceptual Loss

Inspired by human perception, human beings can receive the color and texture information conveyed by the image surface and understand the deep semantic information in the image. So we adopt the perception loss [47] to guide the generator to generate the image more in line with human perception.

(10) $$L_{perc}=E[\sum_{i}\|\Phi_{i}\left(I_{out}\right)-\Phi_{i}\left(I_{gt}\right){\|}_{1}].$$

where $\Phi_{i}$ is the activation map of the *i* layer of the VGG-16 network pre-trained on ImageNet, the corresponding layers to $\Phi_{i}$ in this paper are relu1_1, relu2_1, relu3_1, relu4_1, and relu5_1.

#### 3.3.5. Style Loss

Further, with the perception loss, we adopt the style loss [47] to constrain and keep the consistency between the style and the original image.

(11) $$L_{style}=E[\sum_{i}\|G_{j}^{\Phi }\left(I_{out}\right)-G_{j}^{\Phi }\left(I_{gt}\right){\|}_{1}].$$

where $G_{j}^{\Phi }$ is the Gram matrix with a size of $C_{j}\times C_{j}$ constructed by the activation graph, and the activation graph $\Phi_{i}$ comes from the above perceptual loss.

#### 3.3.6. Joint Loss

In a coarse-stage network, the joint loss function is defined as follows:

(12) $$L_{coarse}=\lambda_{fm}L_{fm}+\lambda_{rec}L_{rec}+\lambda_{adv}L_{adv}.$$

In a fine-stage network, the joint loss function is defined as follows:

(13) $$L_{fine}=\lambda_{rec}L_{rec}+\lambda_{adv}L_{adv}+\lambda_{perc}L_{perc}+\lambda_{style}L_{style}.$$

where $\lambda_{fm}$, $\lambda_{rec}$, $\lambda_{adv}$, $\lambda_{perc}$ and $\lambda_{style}$ are the hyperparameters. According to the experience in [6], we set $\lambda_{fm}=1$, $\lambda_{rec}=1$, $\lambda_{adv}=0.1$, $\lambda_{perc}=0.1$, $\lambda_{style}=250$.

#### 3.3.7. Comparison of Loss Function

**Figure 4:**
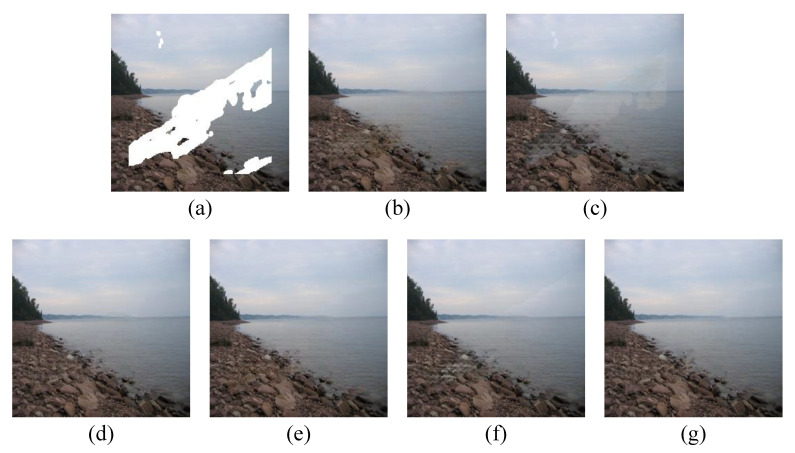
Visual comparison of loss functions. (**a**) Input images. (**b**) W/O feature-matching loss. (**c**) W/O reconstruction loss. (**d**) W/O adversarial loss. (**e**) W/O perceptual loss. (**f**) W/O style loss. (**g**) Result w/ joint loss.

To visually demonstrate the impact of each loss function on image inpainting outcomes, we conducted a comparative experiment for each loss function. As can be seen from Figure 4, the absence of feature-matching loss leads to a deviation in the color and structural features of the object. The removal of reconstruction loss results in a failure of image inpainting. Adversarial loss is crucial for preserving the texture details of the image. Perceptual loss and style loss further enhance the visual effect.

## 4. Experiments

We validate the proposed method on two benchmark datasets: Places2 [48] and CelebA [49]. The damaged image was generated using the mask dataset [30]. The mask dataset contained 12,000 randomly generated mask images. In the experiment, we used four kinds of mask areas as the contrast experiments: 10∼20, 20∼30, 30∼40 and 40∼50. We compare three excellent models to prove the proposed method: GC [13], EC [8], MED [6]. We use random sampling method to extract several categories of images from Place2 for comparative experiments, and the ratio of the training set to test set is 9:1.

### 4.1. Visual Evaluations

**Figure 5:**
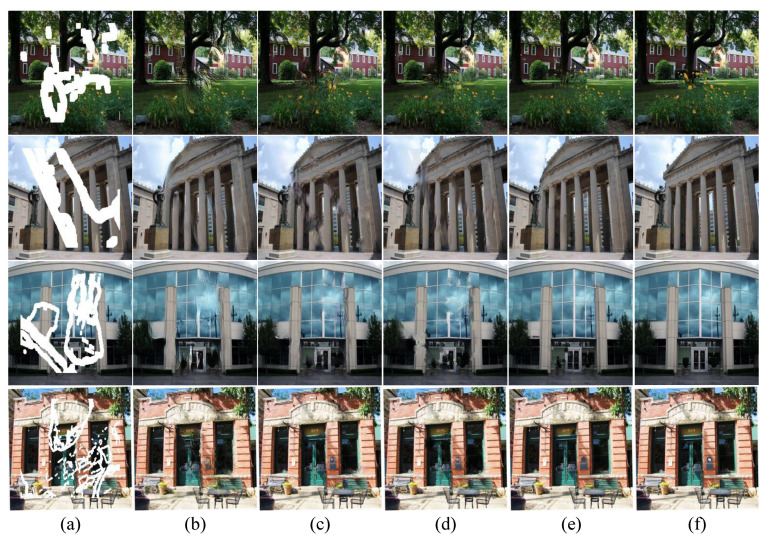
Comparison on the Places2 dataset. (**a**) Input images. (**b**) GC [13]. (**c**) EC [8]. (**d**) MED [6]. (**e**) Ours. (**f**) Ground truth.

**Figure 6:**
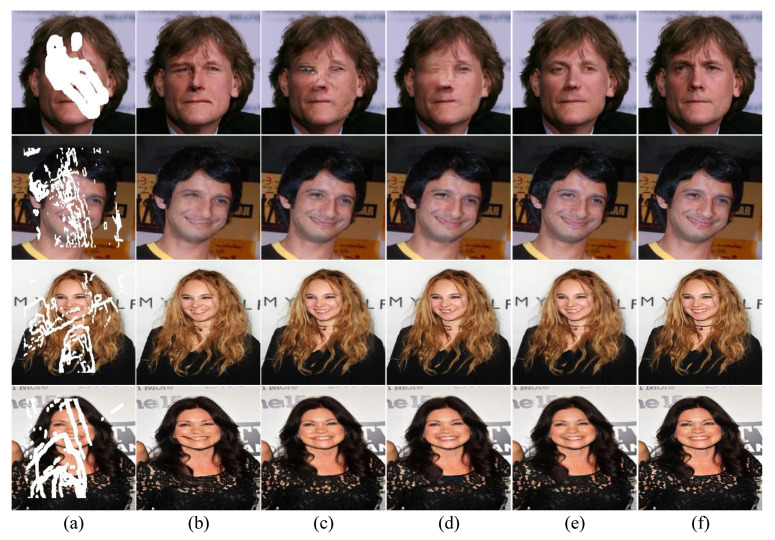
Comparison on the CelebA dataset. (**a**) Input images. (**b**) GC [13]. (**c**) EC [8]. (**d**) MED [6] (**e**) Ours. (**f**) Ground truth.

Figure 5 and Figure 6 show the visual evaluations of the proposed method and the three models on two benchmark datasets. The comparison results on the Places2 dataset show that GC [13] and EC [8], which also use the two-stage generator, often have incorrect semantics, and the structure is too abrupt. MED [6] using a single-stage generator shows such problems as fuzzy texture and inconsistent color. In Figure 5, GC [13], EC [8], and MED [6] fill a large area of damaged image at one time, causing excessive pressure on the generator, which eventually leads to the instability of the restored image, frequent semantic errors. Moreover, the restored facial features lack delicate structure. Our proposed method can effectively solve these problems. The multi-step structure inpainting model is used to reduce the pressure on the generator. The details of the reconstructed image are more exquisite, the texture and color fit the reality, and the additional structural attention module can better fill the structural information, which greatly improves the reliability of the coarse-stage contour reconstruction network. In Figure 5, the first and last lines demonstrate that our method is able to fill in more intricate textures. The second and third lines show that our method effectively reconstructs the structural characteristics of the building without structural errors. In the comparison between the first line and the second line in Figure 6, our method restores more delicate facial features. The third and fourth lines illustrate that our method generates mouth details more realistically.

Experiments show that our multi-step inpainting model can effectively improve the stability of the generator and ensure that the generator can generate images with rich details. The attention mechanism based on the LBP operator can better extract the structural information of the image, strengthen the expression ability of the generator on structural features, and further ensure the inpainting effect in the rough inpainting stage.

### 4.2. Numerical Evaluations

In the numerical evaluation, we used PSNR, SSIM [50] and FID to measure our method and the other three methods. We divided the mask proportion into four groups: 10∼20, 20∼30, 30∼40, 40∼50. Table 1 shows the comparison results of Places2 and Table 2 shows the results of CelebA. The higher the values of PSNR and SSIM, the better the inpainting result, and the lower the value of FID, the higher the similarity with the original image.

The results displayed in the table demonstrate the superior performance of our model compared to other methods on both Places2 and CelebA datasets. The high PSNR and SSIM values indicate that our proposed method generates high-quality images with structural features that are more aligned with reality. The low FID value indicates that the use of a multi-step inpainting model has effectively stabilized the generator and produced inpainted images that are more consistent with the original images. In summary, the multi-step inpainting model and LBP operator-based attention mechanism effectively enhance the inpainting results in the coarse inpainting stage and minimize structural errors in the final inpainted image.

## 5. Ablation Study

In this section, we will continue to validate the proposed method through ablation experiments. We randomly selected a subset of images from the Places2 data set for use in the ablation study. We conducted ablation studies on the multi-step structure inpainting model and the structural attention mechanism to prove the effectiveness of the proposed method.

### 5.1. Multi-Step Structure Inpainting Model

**Figure 7:**
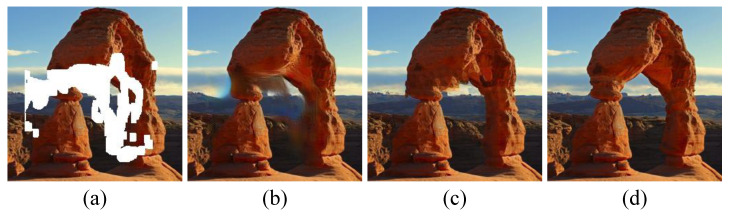
Abalation studies on multi-step structure inpainting model. (**a**) Input image. (**b**) Ours w/o multi-step structure inpainting. (**c**) Ours. (**d**) Ground truth.

To better prove the effectiveness of the method, we control the consistency of other structures and parameters of the model so that whether to enable a multi-step inpainting model is the only variable, and finally verify the proposed multi-step inpainting model on the same mask. As shown in Figure 7, when the entire damaged area is reconstructed at one time, it is difficult to ensure the inpainting effect of the image structure. The resulting image is unstable, and the structure has fractures and semantic errors. The visual effect is improved when we use the multi-step inpainting model, and the generated image conforms to human visual cognition.

### 5.2. Structural Attention Mechanism

**Figure 8:**
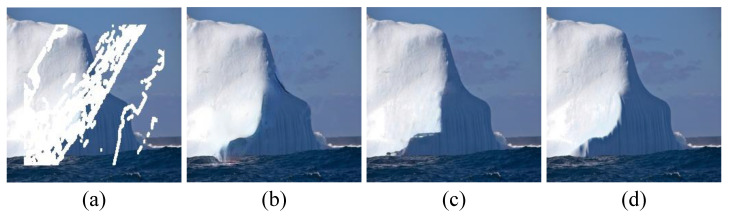
Abalation studies on structural attention mechanism. (**a**) Input image. (**b**) Ours w/o structural attention. (**c**) Ours. (**d**) Ground truth.

In the ablation study of the structural attention mechanism, we control the consistency of other model structures and parameters. The comparison of whether the structural attention mechanism is adopted is shown in Figure 8. We can see from the figure that the structural attention mechanism enhances the structural inpainting of the image and improves the extraction ability of structural features, thus improving the contour reconstruction effect.

## 6. Conclusions

This paper presents a multi-step structure inpainting model to solve the unstable problem of the coarse-to-fine image inpainting model in the first phase. Using the multi-step structure inpainting model reduces the difficulty of image generation, and the effect of image contour rebuilding in the coarse inpainting stage is improved. In addition, we introduced a structure attention mechanism to extract more abundant structure information and enhance the ability to express image structure information. The experimental results on the benchmark dataset show that our proposed method is effective. In future work, we plan to improve the inpainting methods in the fine inpainting stage to fill in finer textures and vivid colors.

## Figures and Tables

**Table 1:** Comparison on the Places2 dataset.

	Mask	GC	EC	MED	Ours
PSNR↑	10∼20	26.580	28.230	28.909	**29.269**
	20∼30	22.551	25.115	25.235	**26.314**
	30∼40	16.485	18.520	19.936	**20.260**
	40∼50	15.130	17.433	17.852	**18.312**
SSIM↑	10∼20	0.909	0.934	0.941	**0.946**
	20∼30	0.754	0.881	0.893	**0.903**
	30∼40	0.652	0.706	0.719	**0.731**
	40∼50	0.560	0.679	0.698	**0.708**
FID↓	10∼20	24.751	22.947	22.022	**18.465**
	20∼30	32.259	31.518	29.063	**26.973**
	30∼40	46.207	45.170	42.570	**38.585**
	40∼50	62.524	59.960	59.179	**54.080**

**Table 2:** Comparison on the CelebA dataset.

	Mask	GC	EC	MED	Ours
PSNR↑	10∼20	27.979	30.473	30.585	**30.946**
	20∼30	20.459	23.179	27.579	**27.614**
	30∼40	17.658	19.973	20.467	**21.056**
	40∼50	16.209	18.216	19.243	**19.420**
SSIM↑	10∼20	0.742	0.907	0.926	**0.934**
	20∼30	0.694	0.862	0.883	**0.892**
	30∼40	0.607	0.744	0.765	**0.774**
	40∼50	0.554	0.628	0.733	**0.739**
FID↓	10∼20	20.580	19.159	19.491	**16.264**
	20∼30	31.472	29.738	27.071	**26.207**
	30∼40	50.485	47.652	43.357	**43.067**
	40∼50	62.975	62.721	58.946	**57.700**

## Data Availability

Not applicable.
